# Immune recovery uveitis in HIV patients with cytomegalovirus retinitis in the era of HAART therapy—a 5-year study from Singapore

**DOI:** 10.1186/s12348-016-0110-3

**Published:** 2016-11-07

**Authors:** Tun Hang Yeo, Tun Kuan Yeo, Elizabeth P. Wong, Rupesh Agrawal, Stephen C. Teoh

**Affiliations:** 1 National Healthcare Group Eye Institute, Tan Tock Seng Hospital, Singapore, 308433 Singapore; 2 Eagle Eye Centre, Singapore, Singapore

**Keywords:** Immune recovery uveitis, Human immunodeficiency virus, Cytomegalovirus retinitis, Highly active antiretroviral therapy, Immune reconstitution inflammatory syndrome

## Abstract

**Background:**

The aim of this study is to analyse the clinical features of HIV patients with cytomegalovirus retinitis (CMVR) developing immune recovery uveitis (IRU) while on highly active antiretroviral therapy (HAART) and to identify the risk factors, visual outcomes and complications of IRU.

**Results:**

Majority (*n* = 26, 86.7 %) of patients were male, with 76.7 % (*n* = 23) of patients having bilateral disease. Twenty-seven eyes (50.9 %) had both anterior uveitis and vitritis. The median CD4 at IRU was 210 cells/μL (IQR 140–279), with 86.7 % having CD4 >100 cells/μL. The median duration from initiation of HAART to IRU was significantly different between those <50 years old (median 763 days, IQR 174–1872 days) and those ≥50 years old (median 161 days, IQR 84.5–278 days). Fourteen eyes (26.4 %) had loss of one or more Snellen lines visual acuity at 6 months while the rest maintained or improved vision. Complications developed in 21 eyes, with cataract (66.7 %), glaucoma and ocular hypertension (33.3 %) being the most common. The risk of complications was associated with the absolute difference in CD4 counts at IRU and at HAART commencement (*p* = 0.041). Age was also negatively associated with the duration from HAART to IRU (*p* = 0.005, Spearman’s rho coefficient = −0.503).

**Conclusions:**

It is common to have both anterior uveitis and vitritis in IRU. There was a positive association between the increase in CD4 from HIV to IRU diagnoses and the risk of developing complications. Younger patients appeared to develop IRU later than older patients after HAART, suggesting that long-term follow-ups are essential for these patients.

## Background

Immune reconstitution inflammatory syndrome (IRIS) is a group of disorders that has been described in HIV patients who are on highly active antiretroviral therapy (HAART). This phenomenon is associated with a paradoxical or exaggerated reactivation of the immune system in the background of a wide range of pathologies such as Kaposi’s sarcoma, tuberculosis, cryptococcal infection and cytomegalovirus [1].

Immune recovery uveitis (IRU) is one manifestation where immune-deficient patients with prior cytomegalovirus retinitis (CMVR) experience an increase in intraocular inflammation following HAART. It is presumed to be mediated by the recovery of immune responses specific to residual cytomegalovirus (CMV) antigens in the eye [2]. IRU is an important cause of visual morbidity in HIV-infected patients with CMVR in the era of HAART [3]. Although immune recovery associated with HAART has allowed some patients to discontinue specific anti-CMV therapy, the rejuvenated immune response can be associated with chronic sight-threatening inflammation. Visual complications of IRU include cataract, cystoid macular edema (CME), glaucoma and epiretinal membrane formation [4–10]. Reported risk factors include a low CD4 count at the time of initiation of HAART and larger areas of CMVR [3]. With the rising prevalence of HIV patients and greater accessibility and availability of HAART worldwide, IRU is an increasingly important disease entity. We aim to analyse the characteristics of IRU in HIV patients on HAART in Singapore, to identify risk factors for the development of IRU and to determine their visual outcomes.

## Methods

A retrospective analysis of clinical records of 30 consecutive IRU patients presenting to the Eye Clinic at the Communicable Disease Centre (CDC), Singapore, between March 2004 and September 2009 was performed. Ethics approval was obtained from the local ethics review board in accordance with the Declaration of Helsinki.

The CDC is the main site of referral and management for HIV in Singapore. IRU was diagnosed when there is an increase in any form of intraocular inflammation from an inactive state of CMV retinitis secondary to immune recovery after initiation of HAART, as evidenced by an increase in CD4 count by 50 or more cells/μL from the nadir CD4 count to a level of more than 100 cells/μL [3]. Despite a CD4 count of less than 100 cells/μL at diagnosis, a small subset of patients are also deemed to have IRU. These patients would have an increase in intraocular inflammation, which is correlated with a rise in CD4 count that could not be clinically attributed to a reactivation of CMVR.

All of the patients had prior CMV retinitis treated with an intravitreal ganciclovir regimen, as previously described [11]. Two patients were also treated with intravenous ganciclovir, and one received oral valganciclovir. Patients with concomitant infective or other autoimmune causes of inflammation were excluded from this study. All patients underwent Snellen best-corrected visual acuity (BCVA) assessment, tonometry and dilated slit-lamp biomicroscopy and indirect ophthalmoscopy examinations. Clinical data obtained included patient demographics (age, gender and ethnicity); duration between HIV, CMVR and IRU diagnoses; BCVA at presentation and at 6 months; corresponding CD4 counts and CD4/CD8 ratios; duration from initiation of HAART to IRU development; presenting signs and symptoms; CMVR zone and area of involvement; IRU severity and location; and treatment regimes. The location of CMVR lesion in each eye was categorised into three zones. Zone 1 was defined as the area within 1500 μm of the optic nerve or within 3000 μm of the fovea. Zone 2 extended from the borders of zone 1 to the vortex veins, and zone 3 involves the retina peripheral to zone 2 [12]. For patients with CMVR involving multiple zones, the zone nearest to the macula was reported.

Descriptive analysis was performed for demographics, visual acuity, complications and IRU and CMVR characteristics, with categorical variables summarised with frequencies and percentages and continuous variables summarised as either means with standard deviations or medians with ranges. Spearman’s rho was calculated to check for association between CD4 counts and durations. Independent sample *t* test and Mann-Whitney *U* test were used for comparison of age, durations and CD4 counts between patient groups with and without complications, respectively. Logistic regression was used to understand the relationship between variables. A *p* value less than 0.05 was considered to indicate statistical significance. Data analysis was carried out using IBM SPSS Statistics (version 19, IBM Corp., NY, USA).

## Results

### IRU characteristics

Twenty-six patients (86.7 %) were male, and the median age at IRU diagnosis was 49.5 years old (interquartile range (IQR) 41–56 years). Chinese patients (*n* = 25) formed the majority, corresponding to the ethnic distribution of the local population [13]. Only eyes (*n* = 53) with previous CMVR, in this cohort, developed IRU. Twenty-three patients (76.7 %) presented with bilateral IRU. Twenty-seven IRU eyes (50.9 %) presented with both anterior uveitis and vitritis. Twenty-two IRU eyes (41.5 %) presented with anterior uveitis alone, while one eye (1.9 %) presented with only vitritis. The median CD4 count at HIV diagnosis of these patients was 20 cells/μL (IQR 15.0–62.0), while the median CD4 count at IRU diagnosis was 210 cells/μL (IQR 140–279), with 86.7 % having CD4 >100 cells/μL. Two of the four patients with CD4 count less than 100 cells/μL had more than 50 cells/μL increase in CD4 count at IRU diagnosis. The median increase in CD4 at IRU was 155.5 cells/μL (IQR 66.0–228.0). The median duration from HIV diagnosis to initiation of HAART was 100.5 days (IQR 61.0–210.0), while the median duration from initiation of HAART to IRU was 214 days (IQR 118.0–763.0). Thirteen IRU patients (43.3 %) were asymptomatic at presentation. Of the remaining 17 IRU patients, the most common symptom was blurring of vision alone (*n* = 11, 36.7 %) followed by floaters (*n* = 4, 13.3 %). All patients were treated with topical steroids, with eight patients requiring additional sub-tenon steroid injections and two patients receiving oral steroids (Tables 1 and 2).

**Table 1:** IRU characteristics

Number of patients, *n*	30
Gender, *n* (%)	
Male	26 (86.7)
Female	4 (13.3)
Race, *n* (%)	
Chinese	25 (83.3)
Malay	3 (10.0)
Indian	1 (3.3)
Others	1 (3.3)
Age, years; mean (SD)	
At IRU diagnosis	47.9 (10.7)
CD4 count, cells/μL; median (IQR)	
At HAART commencement	20.0 (15.0–62.0)
At IRU diagnosis	210.0 (139.8–289.5)
At CMVR diagnosis	28.5 (10.5–61.8)
Difference from HAART to IRU diagnosis	155.5 (66.0–228.0)
Duration, days; median (IQR)	
From HIV diagnosis to HAART commencement	100.5 (61.0–210.0)
From HAART initiation to IRU diagnosis	214.0 (118.0–763.0)
From CMVR diagnosis to IRU diagnosis	136.5 (26.0–618.5)

**Table 2:** IRU-presenting characteristics

Number of patients, *n*	30
Symptoms, *n* (%)	
Blurring of vision	11 (36.7)
Floaters	4 (13.3)
Pain	1 (3.3)
Blurring of vision and floaters	1 (3.3)
None	13 (43.3)
Number of IRU eyes, *n*	53
Presenting features, *n* (%)	
Anterior uveitis alone	22 (41.5)
Vitritis alone	1 (1.9)
Anterior uveitis and vitritis	27 (50.9)
Anterior uveitis and vitreous haemorrhage	2 (3.8)
Vasculitis	1 (1.9)

### CMVR characteristics

All of the patients had CMVR in at least one eye. Fifty-three of the 60 eyes (88.3 %) had previous CMVR. The median duration from HIV diagnosis to CMVR was 283.0 days (IQR 142.0–539.5), while the median duration from CMVR diagnosis to anti-CMVR treatment was 0 days (range −2 to 16), with 24 patients receiving treatment on the same day as CMVR diagnosis. The median CD4 count at CMVR diagnosis in our patients was 28.5 cells/μL (IQR 11.0–60.5). Most eyes (*n* = 23, 43.4 %) had previous CMVR involvement in zone 2. This was followed by zone 1 CMVR involvement in 15 eyes (28.3 %) and zone 3 involvement in 11 eyes (20.8 %). The majority of eyes (*n* = 40, 75.5 %) had <25 % area of involvement, while nine eyes (17.0 %) had 25–50 % area of involvement and four eyes (7.5 %) had >50 % area of involvement (Table 3).

**Table 3:** CMV characteristics

Number of eyes, *n*	53
Location, *n* (%)	
Zone 1	15 (28.3)
Zone 2	23 (43.4)
Zone 3	11 (20.8)
CMVR involvement retinal area, *n* (%)	
<25 %	40 (75.5)
25–50 %	9 (17.0)
>50 %	4 (7.5)
Duration, days; median (IQR)	
From HIV diagnosis to CMVR diagnosis	283.0 (142.0–539.5)
From CMVR diagnosis to anti-CMVR treatment	0.0 (0.0–0.0)

### Visual outcome

The BCVA of IRU patients was assessed at diagnosis of IRU and at 6 months. The range of visual acuity at presentation was 6/4 to NPL, with 19 eyes (35.8 %) having BCVA worse than 6/12. At 6 months, 18 eyes (34 %) had BCVA worse than 6/12. Six (11.3 %) and two eyes (3.8 %) had a loss of one and two Snellen lines, respectively. Six eyes (11.3 %) had a loss of three Snellen lines or more. These were due to glaucoma, cataract or persistent inflammation. The remaining patients had visual acuities that were maintained or better than at IRU presentation (Table 4).

**Table 4:** Visual outcome

Number of affected eyes, *n*	53
Range of VA at IRU presentation, *n* (%)	
6/6 to 6/12	34 (64.2 %)
6/15 to 6/45	12 (22.6 %)
6/60 or worse	7 (13.2 %)
Range of VA at 6-month follow-up post IRU diagnosis, *n* (%)	
6/6 to 6/12	35 (66.0 %)
6/15 to 6/45	10 (18.9 %)
6/60 or worse	8 (15.1 %)
VA change from IRU diagnosis to 6-month follow-up, *n* (%)	
Worsen by at least 3 lines	6 (11.3 %)
Worsen by 2 lines	2 (3.8 %)
Worsen by 1 line	6 (11.3 %)
No change	18 (34.0 %)
Improved by 1 line	7 (13.2 %)
Improved by 2 lines	6 (11.3 %)
Improved by at least 3 lines	8 (15.1 %)

### Complications

Twenty-one eyes (39.6 %) developed complications. The most common complication was cataract (*n* = 14, 66.7 %), followed by glaucoma and ocular hypertension (*n* = 7, 33.3 %). There were no cases of CME during the course of this study. Mann-Whitney *U* test showed an association between the presence of complications and the absolute difference in CD4 counts at IRU and at HAART commencement (*p* = 0.041). For every 50 cells/μL increase in CD4 at IRU diagnosis, patients were 1.28 times more likely to develop complications (*p* = 0.084). Although not statistically significant, the higher CD4 count at IRU diagnosis also appeared to be associated with the development of complications (*p* = 0.057). CMVR characteristics (zone and area of involvement) were not found to be associated with the development of complications (*p* = 0.534 and *p* = 0.715, respectively).

### Risk factors

Age was found to be negatively associated with the duration taken to develop IRU after initiation of HAART (*p* = 0.005, Spearman’s rho coefficient = −0.503). Median duration from HAART initiation to development of IRU was 763 days (IQR 174–1872 days) and 161 days (IQR 84.5–278 days) for patients <50 years old and patients ≥50 years old, respectively. The CD4 counts at HIV diagnosis, at initiation of HAART and at IRU diagnosis were not found to be significantly associated with the duration from HAART to IRU diagnosis. Both the absolute and the percentage increase in CD4 counts were also not significantly associated with the duration taken to develop IRU after initiation of HAART. CMVR zone or area of involvement was not found to be associated with the duration of IRU development from initiation of HAART.

## Discussion

HIV-related mortality has decreased dramatically in the era of HAART. However, it is now evident that the commencement of HAART can also potentially lead to considerable morbidity and mortality in the form of IRIS, especially in the first 6 months [14]. IRU is an important presentation in this broad disease group which also includes tuberculosis-associated IRIS and Kaposi’s sarcoma IRIS [1]. This is the first paper discussing the characteristics of IRU and its complications in Singapore and Southeast Asia. As the Communicable Disease Centre in Singapore manages and treats the majority of local HIV patients [15], our patient cohort is highly reflective of IRU diagnosed locally. The number of HIV-positive patients in Singapore has been gradually increasing, with 454 newly diagnosed cases in 2013 and a total of 6229 patients diagnosed since 1985 [16]. The number of patients with CMVR has increased correspondingly, with 224 patients diagnosed at the CDC from 2005 to 2010 [17]. IRU is most commonly associated with previous CMVR in patients who have experienced immune recovery.

Similar to previous studies, vitritis is the most common presentation in our cohort [5, 10, 18, 19]. There are also a significant proportion of patients who had anterior uveitis. Although anterior uveitis is commonly reported [5, 9, 10, 20], most studies have focused their diagnosis of IRU on the presence of vitritis and associated CME [4, 6, 9, 18, 19].

Patients with larger areas of CMVR involvement were reported to have higher risk of IRU development [9]. In contrast, the majority of eyes in our cohort had <25 % area of previous CMVR involvement. Arevalo et al. did not find an association between CMVR area and risk of IRU development [21]. This suggests that small CMVR lesions are common and eyes without large areas of CMVR invovement are similarly at risk of developing IRU.

Within our cohort, we did not find any association between CD4 counts at HIV diagnosis, at initiation of HAART or at IRU diagnosis, with the development of IRU. However, the duration from the initiation of HAART to IRU was found to be longer in younger patients (<50 years old). Hartigan-O’Connor et al. previously reported that IRU is most likely to develop in patients with the greatest degree of immune dysfunction prior to HAART [22]. Additionally, older age groups have been correlated with quicker seroconversion to AIDS in the pre-HAART era, as well as higher rates of immunologic failure and persistent HIV viraemia in the HAART era [23]. Thus, it is plausible that younger HIV patients have a more intact immune system that may not have such heightened sensitivity to CD4 T cell responses, consequently delaying their development of IRU until the occurrence of further immune dysfunction.

The most common complications of IRU in our cohort were cataract formation, glaucoma and ocular hypertension. Our findings differ from other reports where CME and epiretinal membrane were the most common complications. For instance, Robinson et al. reported that of the IRU eyes in their cohort, 91 % developed CME, 52 % developed cataract and 30 % developed epiretinal membrane [5]. Cataract, CME and epiretinal membrane typically caused moderate visual loss [3, 5, 7].

No cases of CME were reported during the course of this study. Although CME is often described and is a serious sight-threatening complication of IRU, we believe that it is not a common feature, especially if the inflammation is treated earlier. This is particularly pertinent as there is currently no consensus on the ideal time to start treatment of IRU. In fact, the majority of studies have suggested that mild and asymptomatic ocular inflammation usually resolves without treatment; treatment is instituted in more severe inflammation and in patients who become symptomatic [6, 8].

IRU has been described as a transient form of inflammation [19]. Still, others report that the persistence of inflammation in IRU is the cause of complications like CME and epiretinal membrane [6, 18]. Based on our findings, we propose the early treatment of ocular inflammation in IRU patients to prevent sight-threatening complications of CME and epiretinal membrane. Nonetheless, the judicious use of topical steroids in treating asymptomatic patients must be balanced against the possible complications of steroid use.

The risk of IRU complications was found to be higher with greater CD4 count increase from HIV to IRU diagnosis. The greater inflammatory response mounted in these immune reconstituted patients, together with a robust increase in the number of viable T lymphocytes, results in severe and chronic inflammation [24]. The increased inflammatory response can lead to cataract formation, raised intraocular pressure and glaucoma. Conversely, the development of cataract and raised intraocular pressure may also be the result of aggressive steroid therapy, which is often instituted in these patients. However, the mode of steroid therapy did not appear to be associated with the risk of development of complications in this study.

The presence of IRU at baseline was reported to be associated with an increased risk of moderate vision loss to 6/15 [3]. The visual outcome of our IRU patient cohort was, however, fair with only eight eyes losing two or more lines of Snellen VA. 73.6 % of eyes (*n* = 39) had either maintained or improved in vision. The importance of early diagnosis with aggressive and appropriate treatment can improve and preserve vision. Moreover, a number of patients were asymptomatic and were found to have IRU only on routine follow-up examinations. All HIV patients with treated CMVR should continue to have regular reviews at the eye clinic at regular intervals, even if they have high CD4 counts. Our study showed the median time from commencement of HAART to IRU development to be about 7 months. The longest time taken was 8.8 years in a 25-year-old female. Without regular follow-up, early recognition and treatment, the visual outcomes of these patients may be less favourable.

The main limitations of this study were its retrospective design and relatively small sample size. There was also no control group. Unlike other studies, we did not routinely perform angiogram on our patients. Hence, it is possible that early CME not apparent on physical examination was missed. Nonetheless, our cohort of patients was diagnosed consecutively from the CDC, which is reflective of the HIV patient profile in Singapore, as the CDC is the main centre for HIV treatment.

## Conclusions

IRU is common and usually presents with vitritis. Early treatment of ocular inflammation can prevent the development of sight-threatening complications of IRU. The most common complication is cataract formation, and the risk of complications was positively associated with the increase in CD4 count at IRU and HIV diagnosis. Younger patients also appeared to develop IRU later than older patients after HAART, emphasising that long-term follow-ups are essential for these patients.
